# Impacts of a comprehensive tuberculosis control model on the quality of clinical services and the financial burden of treatment for patients with drug-resistant tuberculosis in China: a mixed-methods evaluation

**DOI:** 10.1186/s40249-021-00832-5

**Published:** 2021-04-21

**Authors:** Wei-Xi Jiang, Zhi-Peng Li, Qi Zhao, Meng-Qiu Gao, Qian Long, Wei-Bing Wang, Fei Huang, Ni Wang, Sheng-Lan Tang

**Affiliations:** 1 Global Health Research Center, Duke Kunshan University, No. 8 Duke Avenue, Kunshan, 215316 Jiangsu China; 2 School of Public Health, Fudan University, 130 Dongan Road, Shanghai, 200032 China; 3 Beijing Chest Hospital, Capital Medical University, No. 9 Beiguan Road, Beijing, 101149 China; 4 National Center for Tuberculosis Control and Prevention, China CDC, No.155 Changbai Road, Changping District, Beijing, 102206 China; 5 National Center for Tuberculosis Control and Prevention, China CDC, No.27 Nanwei Road, Xicheng District, Beijing, 100050 China; 6 Duke Global Health Institute, Duke University, 310 Trent Drive, Durham, NC 27710 USA

**Keywords:** Drug-resistant tuberculosis, Quality of health services, Financial burden

## Abstract

**Background:**

The China National Health Commission-Gates TB Project Phase III implemented a comprehensive TB control model including multiple interventions to address the burden of drug-resistant TB (DRTB). This study aims to evaluate the quality of DRTB clinical services and assess the financial burden of DRTB patients during the intervention period.

**Methods:**

A mixed-methods approach was used to evaluate the effectiveness of interventions in the three project provinces: Zhejiang, Jilin and Ningxia Hui Autonomous Region. The quantitative data included de-identified DRTB registry data during 2015–2018 in project provinces from China CDC, medical records of DRTB patients registered in 2018 (*n* = 106) from designated hospitals, and a structured DRTB patient survey in six sample prefectures in 2019. The quality of clinical services was evaluated using seven indicators across patient screening, diagnosis and treatment. Logistic regression was conducted to explore factors associated with the extremely high financial burden. Semi-structured in-depth interviews with policymakers and focus group discussions with physicians and DRTB patients were conducted to understand the interventions implemented and their impacts.

**Results:**

The percentage of bacterially confirmed patients taking a drug susceptibility test (DST) increased significantly between 2015 and 2018: from 57.4 to 93.6% in Zhejiang, 12.5 to 86.5% in Jilin, and 29.7 to 91.4% in Ningxia. The treatment enrollment rate among diagnosed DRTB patients also increased significantly and varied from 73 to 82% in the three provinces in 2018. Over 90% of patients in Zhejiang and Jilin and 75% in Ningxia remained in treatment by the end of the first six months’ treatment. Among all survey respondents 77.5% incurred extremely high financial burden of treatment. Qualitative results showed that interventions on promoting rapid DST technologies and patient referral were successfully implemented, but the new financing policies for reducing patients’ financial burden were not implemented as planned.

**Conclusions:**

The quality of DRTB related clinical services has been significantly improved following the comprehensive interventions, while the financial burden of DRTB patients remains high due to the delay in implementing financing policies. Stronger political commitment and leadership are required for multi-channel financing to provide additional financial support to DRTB patients.

**Figure:**
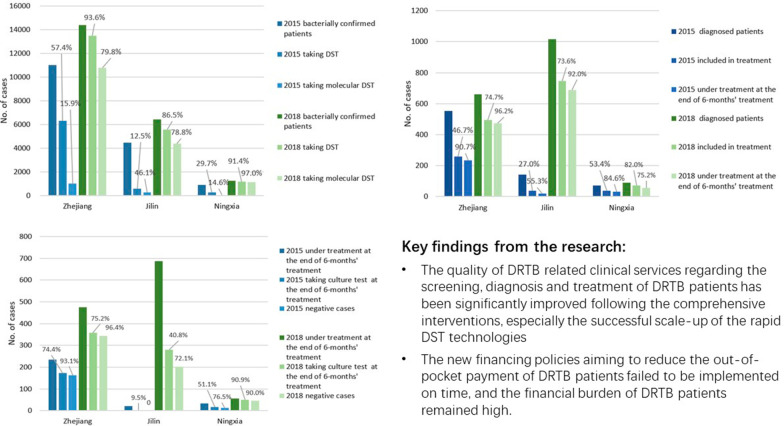

**Supplementary Information:**

The online version contains supplementary material available at 10.1186/s40249-021-00832-5.

## Background

Drug-resistant tuberculosis (DRTB) presents a new challenge to bringing an end to TB globally [1]. According to the World Health Organization (WHO), 3.3% of new TB patients and 18% of previously treated patients developed rifampicin-resistant TB (RRTB) in 2019, including multi-drug resistant TB (MDR-TB). China has a high burden of DRTB: it made up 14% of the total RR/MDR-TB cases in 2019. China together with India also accounted for 41% of global gaps between the number of RR/MDR-TB patients enrolled in treatment and the estimated number of new cases as reported by the WHO [2]. There is an urgent need for effective DRTB control strategies and policies to achieve the Sustainable Development Goal of ending the TB epidemic by 2030 in China [3].

Previous studies in many low-and-middle income countries, including China, have revealed several barriers to improving case detection and treatment of DRTB. The diagnosis of DRTB takes around 2 months using traditional technologies, such as a culture test. The long waiting time for diagnosis has largely contributed to pre-diagnosis dropout of patients, especially for poor patients who exhaust available funds during pre-diagnosis treatment [4–6]. Besides, the standard treatment for RR/MDR-TB patients is expensive and time-consuming, and the accessibility of quality treatment has been challenging in developing countries [7, 8]. In China the financial protection provided by the three types of public health insurance in China, [the Urban Employee Basic Medical Insurance (UEBMI), Urban Resident Basic Medical Insurance (URBMI), and new rural cooperative medical schemes (NCMS)], is often insufficient to cover the costs. One major reason is that many medical services, such as some second-line anti-TB drugs, are only partially covered by insurance or may need to be paid fully out-of-pocket [9–12]. Additionally, under China’s current TB control model, DRTB patients are treated in the prefecture-level designated hospital, typically a third-level hospital with relative low reimbursement rates under the public health insurance system. Therefore, the standard treatment is often unaffordable to many DRTB patients and consequently, patients either default treatment or sell their properties or borrow money to cope with the treatment cost, and become poverty-stricken [13–16]. Poor adherence to treatment may also influence the treatment outcome. According to the WHO, the treatment success rate for RR/MDR-TB patients in China who started on second-line treatment was 54% in 2017 [17].

A number of interventions have proven effective in terms of addressing these challenges in accessing the clinical services for presumptive and diagnosed DRTB patients. Rapid drug susceptibility test (DST) technologies, such as GeneXpert, can provide DST result for rifampicin in a single day. Rapid DST has been proved effective in improving case detection, thereby reducing the likelihood of pre-diagnosis attrition [18–21]. Other interventions focusing on the management of DRTB patients, such as directly observed therapy, patient-centered support, and the use of e-health technologies, could also improve treatment adherence of MDR-TB patients [22, 23]. The evidence on the effectiveness of providing additional financial support on the accessibility and outcomes of DRTB treatment is relatively scarce, though many international donor-sponsored TB control projects involve economic support for patients [24]. Nevertheless, one comprehensive program providing universal access to health care for MDR-TB patients improved the treatment initiation rate and adherence to treatment, and significantly decreased out-of-pocket (OOP) medical expenditures [25]. Another study also found that psychological and financial support provided together could improve the cure rates for MDRTB patients [26].

To address TB control challenges in China, the China National Health Commission (NHC)-Gates TB Project Phase III has implemented a new comprehensive model of TB control in three Provinces: Zhejiang, Jilin, and the Ningxia Hui Autonomous Region. Based on experiences from previous phases as well as other scientific evidences, the model for Phase III included a set of interventions aimed at (1) improving the accessibility and quality of DRTB-related health services and (2) relieving the financial burden of DRTB patients. According to the intervention design, rapid test facility such as GeneXpert would be equipped at both the prefecture and county-level TB designated hospitals for DRTB screening, and the supply of reagents would also be guaranteed through supporting policies. Besides, the bi-directional referral policies require that the presumptive DRTB patients be transferred to the prefecture-level hospital for diagnosis and standard treatment. Multiple financing resources, including both public health insurance and government specialized funding, would also be mobilized to provide additional financial protection for DRTB patients and reduce the percentage of out-of-pocket (OOP) medical expenditures to 10%. As part of the project evaluation, this study aims to assess whether the designed interventions were successfully implemented in the project provinces and investigate the impacts of these interventions on the clinical performance of DRTB related health care services in terms of case detection and treatment, and the financial burden of DRTB patients during the intervention period.

## Methods

### Study sites

The study was conducted in the three project provinces where the Gates TB Project Phase III was implemented: Zhejiang, Jilin, and the Ningxia Hui Autonomous Region ((herein referred to as ZJ, JL and NX), and these sites were selected according to their range in socioeconomic development in order to examine the effects of interventions in different resource settings. ZJ is the most developed province in eastern China with an annual disposable per capita income of CNY 45 840, ranking 3rd among the 31 provinces in 2018. JL and NX are less developed than ZJ, ranking 18th and 22nd respectively [27]. Under the TB prevention control model in ZJ and JL, the anti-DRTB treatment should be provided by the prefecture-level designated medical institutions, usually comprehensive third-level hospitals in ZJ and specialized infectious disease hospitals or TB dispensaries in JL. In NX only the provincial level hospital, an infectious disease hospital, provides standard anti-DRTB treatment. In addition to the data collection at the provincial level, we also conducted in-depth evaluations in six sample prefectures, two in each province. We selected one prefecture with a relatively high level of economic development and one with a lower level of economic development in each province.

### Study design

This study used a mixed-methods approach. We combine quantitative analyses of DRTB case records from the Tuberculosis Information Management System (TBIMS), medical records from DRTB designated hospitals, and data from a DRTB patient survey as well as qualitative research including semi-structured in-depth interviews with key informants and focus group discussions (FGDs) with physicians and patients on the clinical service for and financial burden of DRTB.

### Data collection

#### TBIMS records and medical records

The de-identified dataset of DRTB patients registered between 2015 and 2018 in ZJ, JL and NX was retrieved from the TBIMS of Chinese Center for Disease Control and Prevention (China CDC). While previously only MDR/XDR-TB patients were registered as a specialized category in TBIMS, in recent years patients resistant to at least one type of first-line anti-TB drug are gradually registered and included in standardized anti-DRTB treatment according to their DST results. The dataset contained three basic sets of information: (1) demographic information of DRTB patients including age, gender, ethnicity, residence, etc.; (2) diagnosis and treatment information, including all drug-resistant tests (DST) taken, the results, and the status of any anti-DRTB treatment; and (3) sputum and culture tests taken during the treatment period.

The medical records of all DRTB patients registered in 2018 in the six sample prefectures who had completed inpatient treatment were retrieved from designated hospital. In total 106 medical records were examined: 24 in ZJ, 58 in JL, and 22 in NX. These records were examined by clinical experts according to the following constraints: (1) the usage of molecular DST; (2) the rationality of treatment regimen, defined as whether drugs in the prescription were selected based on DST results using the guidelines developed by China CDC; and (3) the treatment status and the negative rate of culture test after 6-months’ treatment. Similar examination was also conducted at the baseline survey in 2015, but the sample size (< 50) was too small for the comparison before and after intervention.

#### DRTB patient survey

The DRTB patient survey was conducted in the six sample prefectures in August 2019. The questionnaire collected information on patient demographic and socio-economic status, treatment experiences, treatment costs, and OOP expenditures during the first six months of treatment. Eligible participants include DRTB patients that were registered between February 1, 2017 and February 1, 2019 in the six study sites who had completed six months of treatment. Patients over 15 years old were sampled successively following a reverse chronological order of the registration date. The expected sample for each prefecture was 70, based on the number of patient cases in the study period. If the total number of eligible patients was less than 70, then all patients were sampled. Face-to-face interviews were conducted by trained investigators using a structured survey questionnaire loaded in a tablet PC. Oral consent was obtained before the interviews.

#### Qualitative data

Semi-structured in-depth interviews were conducted to understand the how the designed interventions were implemented during the project period. In-depth interviews were conducted with informants from provincial and prefectural level health commission (*n* = 9), CDC (*n* = 9), health insurance office (*n* = 9) and the insurance division of designated hospital of the sample prefectures (*n* = 9).

Focus group discussions were conducted to understand patient treatment experiences and causes of financial burden. FGDs were held with physicians in designated hospitals (*n* = 7) and DRTB patients (*n* = 6), and each focus group had 4–6 participants. Eligible DRTB patients were those who had completed 6 months of treatment. Patients were selected based on their gender, socio-economic status, insurance type, and distance from downtown areas.

All interviews and FGDs were conducted in a private room by experienced researchers. Oral consent was obtained before tape-recording. On average, each interview lasted 30–45 min and the FGD was around one hour.

### Data analysis

The quality of clinical service for DRTB patients was evaluated in the three areas: (1) patient screening and diagnosis, including the percentage of bacteriologically positive TB patients ever taking DST and molecular DST; (2) treatment status, including the percentage of patients included in treatment and remain under treatment 6 months after treatment initiation; (3) rationality and outcome of treatment, including the percentage of patients taking the culture test, the percentage of patients with negative culture test results after six months of treatment, and the proportion of rational regimen (Table 1). Indicators a–f were calculated using the TBIMS records for patients in 2015 as the baseline, and in 2018 as the final evaluation. Chi-square tests were conducted to see if there were significant differences between the baseline and the final. Data from the examination of the medical records were also used to calculate indicators d–g. Results from the two resources on the same indicator would also be cross-checked for validation, and were reported by province.

**Table 1:** Key indicators for evaluating the quality of clinical service for drug-resistant TB patients

Area of clinical quality	Indicator
Screening and diagnosis	a. The percentage of bacteriologically positive TB patients ever taking DST
	b. The percentage of bacteriologically positive TB patients ever taking molecular DST
Treatment status	c. The percentage of patients included in treatment
	d. The percentage of patients under treatment at the end of 6th months’ treatment among those treated
Rationality and outcome of treatment	e. The percentage of patients taking culture test after 6 months’ treatment
	f. The case negative rate among those taking culture test after 6 months’ treatment
	g. The proportion of rational regimen of all examined medical records

*TB* tuberculosis, *DST* drug susceptibility test

For the patient survey, descriptive analysis was conducted to understand basic patient characteristics. Among the 204 respondents of the survey, 147 (72.1%) reported OOP medical expenditures. Therefore, we compared the characteristics of those patients who reported OOP expenditures with those who did not using a Chi-squared test. A multiple imputation (MI) method was used to address missing data in the self-reported OOP medical expenditures, and the analysis of the financial burden were based on the results of MI [28]. Self-reported OOP medical expenditures were calculated for the first six months of treatment. The financial burden of patients was evaluated using the three thresholds of incurring catastrophic medical expenditure, defined as OOP medical expenditure exceeding 10%, 20% and 30% of annual household income respectively [29]. Logistic regression was conducted to explore factors associated with the extremely high financial burden of treatment (defined as OOP medical expenditure ≥ 30% of annual household income).

Qualitative data was analyzed using a thematic analysis approach [30]. The analytic framework was developed based on the topic guides, and was refined regarding the emerging themes during the coding process. All qualitative data were coded and classified under each theme of the analytic framework, and we compared findings by different stakeholders to identify commonality and divergent perceptions. We analyzed the original Chinese texts and translated the results to English.

## Results

### Changes in the quality of clinical services

#### Screening and diagnosis

DRTB patient records in TBIMS show that the number of diagnosed patients increased drastically from 766 in 2015 to 1765 in 2018. Specifically, the number of diagnosed DRTB patients increased sixfold in JL, from 141 in 2015 to 1016 in 2018. Compared to 2015, DRTB patients in 2018 tended to be older in age, and there were higher percentage of patients living in the county of registered residence. Among all registered patients, about 73% were male, and the ethnicity other than Han was primarily the Hui Nationality in NX (see Additional file 1: Table S1).

**Fig. 1:**
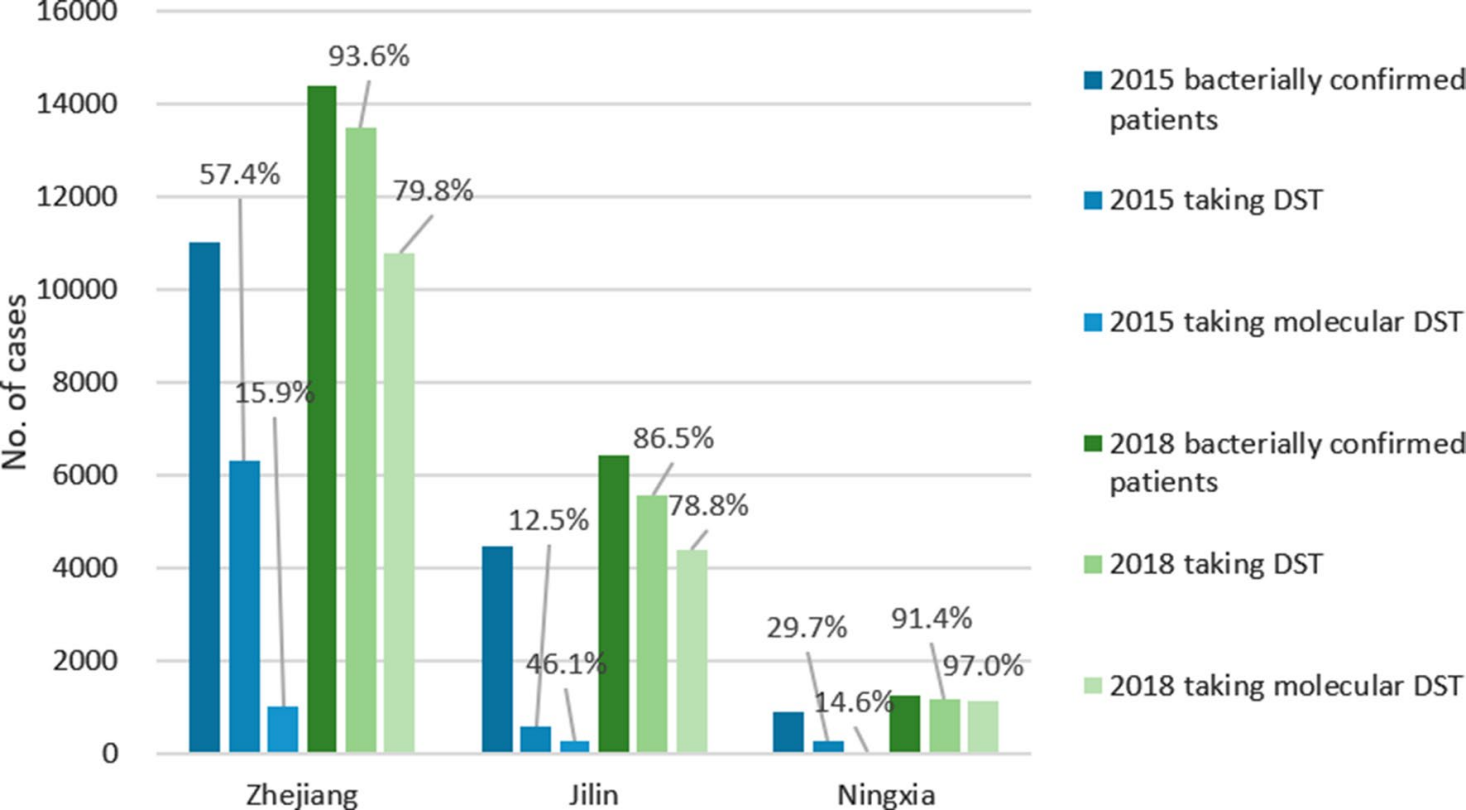
The number and percentage of bacterially confirmed patients taking drug susceptibility test at baseline and final evaluation

Results from the TBIMS data show that the percentage of bacterially confirmed patients taking DST increased significantly in all three project provinces (Fig. 1). Prior to project implementation in 2015, the proportion was only 12.5% in JL, 29.7% in NX, and 57.4% in ZJ. Despite varying performances prior to the project, by 2018, the proportion in each province was 86.5%, 91.4%, and 93.6% respectively. The proportion of bacterially confirmed patients taking molecular DST among those tested also increased from less than 50% at baseline in all three provinces, to 79.8% in ZJ, 78.8% in JL and 90.0% in NX (see Additional file 1: Table S2 for detailed figures and results of Chi-square tests).

Qualitative studies revealed that successful scale-up of the new rapid DST technologies, mainly GeneXpert, contributed to the increase of DRTB case finding. Most key informants indicated that the successful promotion of the new DST technologies relied on government funding to guarantee the reagents for the assays and provide the screening test free-of-charge for presumptive DRTB patients, thus ensuring the use of the rapid DST technologies when necessary. Nevertheless, some physicians expressed concern that the funding support for reagents could not be sustained after the end of the project. As a way to mitigate such sustainability issues, they proposed that the government negotiate with suppliers to lower the price of rapid DST and encourage its inclusion in the benefit package of public health insurance schemes.

**Table:** Box 1. Quotations on the scale-up of rapid drug susceptibility test technologies

*We began to use GeneXpert to conduct the screening test for all active TB patients since September, 2017, and now it is free-of-charge for patients, and the provincial-level designated hospital would provide the reagents. (in-depth interview, prefecture-level hospital, Ningxia)* *Since September, 2018 all counties in Jilin could conduct molecular DST regularly. 38 of the counties have equipped GeneXpert, and the rest counties used GeneChip or other technologies…(in-depth interview, Jilin academy of TB prevention and treatment)* *Every county in our province had equipped GeneXpert, and 73% of the province had GeneXpert and fluid culture facilities at the same time…GeneXpert testing is free of charge for TB patients. (in-depth interview, CDC, Zhejiang)* *GeneXpert has not been covered by the health insurance yet…Now it is provided free of charge during the project period and we are afraid that after the project if patients have to pay out-of-pocket, they would not choose to take the test. (FGD, designated hospital, Zhejiang)*	

*DST* Drug susceptibility test, *FGD* Focus-group discussion, *CDC* Center for Disease Control and Prevention

#### Treatment status

**Fig. 2:**
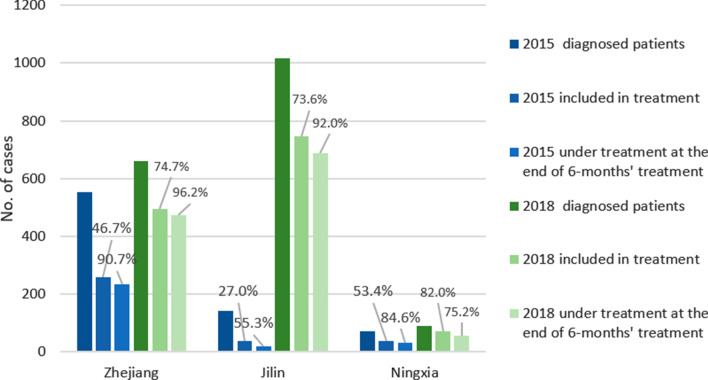
Drug-resistant TB patients diagnosed, included in treatment and under treatment at the end of 6-months’ treatment

Data from the TBIMS records show that the number of DRTB patients included in treatment also increased sharply in all three provinces after the project intervention. The largest increase was in JL, where the number of patients increased nearly 20-folds from 38 to 747. The percentage of diagnosed patients in JL who received treatment rose from 27.0 to 73.6%. In ZJ and NX, this percentage also increased to 74.7% and 82.0% respectively in 2018 (Fig. 2).

Among patients who received treatment, the dramatic increase in the proportion of patients who were still under treatment at the end of the 6 months’ treatment was also seen in JL, from 55.3% in 2015 to 92% in 2018, and the increase was also statistically significant in ZJ, reaching 96.2% in 2018 (see Additional file 1: Table S3 for detailed figures and results of Chi-square tests). Results from onsite examination from medical records on this indicator were similar, around 85% to 90% in the three provinces (see Additional file 1: Table S5).

Qualitative findings on the treatment of DRTB patients revealed how project interventions may help improve the overall quality of DRTB health services. Under the bi-directional referral policies implemented during the intervention period, the presumptive DRTB patients were required to be transferred to prefecture-level designated hospital for diagnosis and treatment from all types of health facilities, thus enrolling patients into standard treatment once diagnosed. Moreover, the supervision on the implementation of these policies by the clinical and public health experts organized by CDC were conducted regularly in all project provinces, and the results were reported across the province to push the designated hospitals to improve their performance.

**Table:** Box 2. Quotations on the bi-directional referral policies and the supervision on the implementation of interventions

*The Bi-directional referral policies have been implemented in around 90% counties of the provinces. The coordination among the (prefectural-level) infectious disease hospital, county-level TB dispensary and the township hospital was smooth. (in-depth interview, health commission, Jilin)* *Our province is small, and the DRTB patients are treated in the provincial level hospital. DRTB patients will be directly transferred from county-level hospital to the provincial level designated hospital. ((in-depth interview, health commission, Ningxia)* *The CDC would organize a team of experts to come (to check our work) once a month, the Health commission also authorized the Ningxia Fourth Hospital to assess our TB clinical service twice a year, and the results will be reported across the province… (in-depth interview, prefecture-level hospital, Ningxia)*	

*DRTB* Drug-resistant tuberculosis, *CDC* Center for Disease Control and Prevention

#### Rationality of treatment regimen and outcome of treatment

**Fig. 3:**
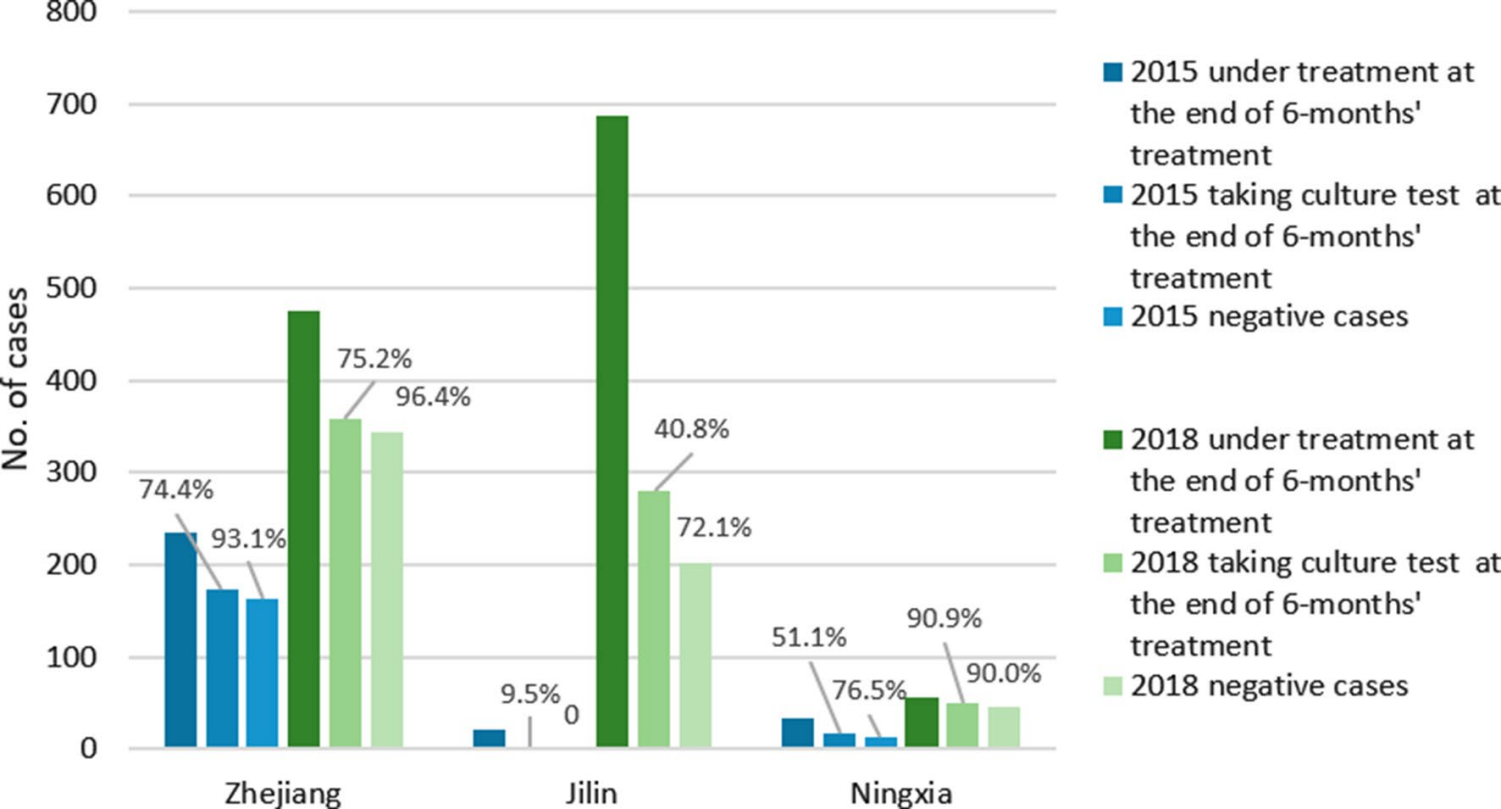
Drug-resistant TB patient taking culture test at the end of 6-months’ treatment and the negative cases

Results from the TBIMS records showed that the percentage of patients taking a culture test among those under treatment at the end of six months’ treatment increased significantly in JL (from 9.5 to 40.8%) and NX (from 51.5 to 90.9%) while the proportion remained the same ZJ (around 75%) (Fig. 3). For JL specifically, the number of DRTB patients taking culture test at the end of the 6 months of treatment increased from 2 to 280 (see Additional file 1: Table S4 for detailed figures and results of Chi-square tests). Results from the medical records on this indicator were also similar, except for NX where the sample size was very small (*n* = 19) and may result in the discrepancy in the percentages (see Additional file 1: Table S5).

For patients who received a culture test, the negative rate of the sample remained above 90% in ZJ and reached 90% in NX. In JL the percentage reached 70%, still a dramatic improvement from 0. Results from the medical records showed higher percentage of negative cases (92.9%), but the sample size was small. Additionally, medical records examinations found that the percentage of rational regimen was 100.0%, 84.5%, and 77.3% in ZJ, JL, and NX respectively. The major problem in the regimen was that the types of drugs were not sufficient, for example, cycloserine oxymycin was often not prescribed when needed.

Qualitative interviews have revealed how the interventions could be effective in standardizing physicians’ clinical practices and improving the overall quality of DRTB-related clinical services. Nevertheless, challenges remain in further improving the clinical quality. Some physicians complained that certain types of second-line anti-TB drugs were restricted by the health insurance, thus they were often not able to prescribe them. Physicians in NX also explained to the experts who examined the medical records that the supply of some second-line drugs was limited, and often in deficiency. Additionally, some patients in less developed regions were not willing to go to prefecture-level or higher-level designated hospitals for treatment as required, for reasons such as perception of high costs or inconvenience.

**Table:** Box 3. Quotations on the challenges to improve quality

We have not received the new formal clinical guideline and thus unable to use it to discuss with the NCMS on the reimbursement policies…Some medicines that could be used by the DRTB patients were restricted by the NCMS, and the physicians would be fined if prescribe those medicines. (in-depth interview, infectious disease hospital, Jilin) Some DRTB patients were not willing to go to Ningxia Fourth Hospital because it was to faraway for them…(FGD, prefecture-level hospital, Ningxia)	

*NCMS* New Cooperative Medical Schemes, *DRTB* Drug-resistant tuberculosis, *FGD* Focus-group discussion

### Financial burden of DRTB patients

#### Patient characteristics

As shown in Table 2, a majority of the patients who participated in the survey were from JL and 27% were female, almost the same as of the gender distribution of the registered. The percentages of middle-aged patients and those who resided in the county of registered residence (Hukou) were higher for the survey respondents compared to all registered patients in the three provinces (see Additional file 1: Table S1). MDRTB patients were also likely to be the disadvantaged social group as almost 60% were from rural area, around 2/3 never attending high school, and 1/3 of the patients’ household income per capita were below the national poverty line. As for the comparison between patients with or without data on OOP medical expenditure, results show that female and patients with lowest education level were less likely to report OOP. (*P* < 0.05, see Additional file 1: Table S6).

**Table 2:** Characteristics of the participants of the patient survey

	*N*	%
Total	204	/
Province		
Zhejiang	39	19.1
Jilin	120	58.8
Ningxia	45	22.1
Gender		
Female	55	27.0
Male	149	73.0
Age		
< 30	29	15.3
30–59	118	62.4
> 59	42	22.2
Ethnicity		
Han	162	85.7
Other	27	14.3
Marriage		
Married	133	65.2
Current place of residence		
In the county of registration	179	87.8
In the prefecture of registration, but not the county	12	5.9
In the province of registration, but not the prefecture	0	0.0
Outside the province of registration	13	6.4
Type of registered residence		
Rural	121	59.3
Education level		
Primary school and below	66	32.4
Junior high school	66	32.4
Senior high school and the equivalent	41	20.1
College and above	31	15.2
Insurance coverage		
UEBMI	38	18.6
NCMS	93	45.6
URBMI	62	30.4
Other	11	5.4
Household income per capita		
Below CNY 3747 (poverty line in 2019)	67	33.5
NCD status		
With other NCD	76	37.3

*UEBMI* Urban Employee Basic Medical Insurance, *URBMI* Urban Resident Basic Medical Insurance, *NCMS* New Cooperative Medical Schemes, *NCD* non-communicable disease

### Financial burden in the three provinces and the associated factors

Results from the patient survey show that mean OOP medical expenditures in ZJ and JL were CNY 25 910 and CNY 27 816 respectively, which is more than 35% higher than in NX (Table 3). Nevertheless, the mean household income of ZJ patients was 50% higher than patients in JL, and more than double the income of NX patients. The proportion of patients incurring CHE was also lowest in ZJ, and was similar in JL and NX. The financial burden of MDRTB patients was still very high, as even using the highest threshold that OOP medical expenditure exceeding 30% of household income, 77.5% of the patients incurred CHE.

**Table 3:** The out-of-pocket health expenditure and the occurrence of catastrophic health expenditure for the first six months of treatment

Province	Mean medical OOP/CNY	Mean per capita household income/CNY	Mean household income/CNY	% CHE-10%	% CHE-20%	% CHE-30%
Zhejiang	25 910	16 614	49 572	77.0	68.1	62.4
Jilin	27 816	13 206	32 740	94.2	85.0	80.9
Ningxia	19 202	5698	21 691	92.8	87.9	82.6
Total	24 806	12 181	33 536	90.3	82.2	77.5

*OOP* out-of-pocket, *CHE* Catastrophic health expenditure

Results of logistic regression of factors associated with incurring extremely high financial burden for treatment are shown in Table 4. After adjusting for other demographic and socio-economic factors, patients in JL were four times more likely to incur extremely high financial burden from treatment than those in ZJ (95% *CI* 1.01–16.35). Patients over the age of 59 were much less likely to incur this high financial burden (95% *CI* 0.00–0.97), probably due to the low willingness to pay for treatment. Patients in the highest income group were less likely to incur high financial burden compared to the poorest group (95% *CI* 0.02–0.65). Additionally, local patients, (i.e., those that live in the county of registration) have a lower likelihood of incurring extremely high financial burden for treatment, although this is not statistically significant (*OR* = 0.14, *P* = 0.062).

**Table 4:** Factors associated with incurring extremely high financial burden for drug-resistant TB patients

	*OR*	*P* value	95% *CI*	
Province				
Zhejiang	Ref			
Jilin	4.07	0.048	1.01	16.35
Ningxia	3.51	0.184	0.55	22.38
Gender				
Male	1.40	0.624	0.36	5.47
Age				
< 30	Ref			
30–59	0.16	0.155	0.01	2.02
> 59	0.07	0.047	0.00	0.97
Ethnicity				
Han	2.22	0.449	0.28	17.78
Marriage				
Married	1.07	0.899	0.37	3.10
Current place of residence				
In the county of registration	0.14	0.062	0.02	1.11
Type of registered residence				
Rural	0.41	0.335	0.07	2.55
Education level				
Primary school and below	Ref			
Junior high school	0.77	0.738	0.16	3.71
Senior high school and above	0.64	0.622	0.11	3.88
Insurance coverage				
UEBMI	Ref			
NCMS	4.91	0.109	0.70	34.59
Other	3.16	0.139	0.68	14.63
Average household income				
Lowest 1/3	Ref			
Middle 1/3	0.41	0.330	0.07	2.54
Highest 1/3	0.12	0.015	0.02	0.65
NCD status				
With other NCD	0.58	0.352	0.18	1.86

*UEBMI* Urban Employee Basic Medical Insurance, *NCMS* New Cooperative Medical Schemes, *NCD* non-communicable disease

The interviews with key stakeholders revealed that the new financing policies aiming to limit the percentage of OOP payment were issued in late 2018 and 2019 in JL and NX, and may have not been actually implemented in some places at the time of final evaluation. Therefore, in our research, very few patients in JL and NX ever enjoyed the new policies. Some interviewees in the less-developed regions also expressed concerns over the availability of additional funds due to financial difficulties of the local government. In ZJ there had been some financial assistance policies for DRTB patients to help cover remaining medical expenditures after health insurance reimbursement in 2015.

**Table:** Box 4. Quotations on the new financing policies for DRTB patients

*For MDRTB patients the reimbursement rate for outpatient visit has been improved to the level of inpatient service in 2015…The health insurance covered 70% and the provincial and prefectural financing department covered the rest 30%. (in-depth interview, Zhejiang CDC)* *In July 2018 the government began to estimate the money needed for the new financing policies that set the limit of the percentage of the OOP medical expenditure to 10%. The financing department has passed a plan of 14 million CNY, but the policies had not been implemented yet…(FGD, designated hospital, Jilin)* *The financing policies came out late at the prefecture-level mainly due to the financial difficulties of the government that the funds could not be secured, because the economic development in our province was stumbled in recent years… (in-depth interview, Jilin academy of TB prevention and treatment)* *The new policies was implemented in Oct. 2018 that the DRTB patients pay less than 30% of the total medical costs…The patients use their bank card to pay for the medical cost at the hospital, and we transfer the money to patients in 24 h…(in-depth interview, health insurance office, Ningxia)*	

*MDRTB* Multi-drug resistant tuberculosis, *OOP* out-of-pocket, *FGD* Focus-group discussion, *DRTB* Drug-resistant tuberculosis, *CDC* Center for Disease Control and Prevention

FGDs with physicians and DRTB patients revealed several reasons for the extremely high financial burden during treatment. One major reason is that DRTB patients were treated at prefecture-level designated hospitals or above, usually third-level hospitals, for which the reimbursement rate of public health insurances was relatively low with the absence of new policies. Moreover, many medical services used during the anti-DRTB treatment were not covered by health insurance nor medical assistance. For example, physicians reported that medicines not covered by the public health insurance were restricted to be within 10% of total costs in a prescription as a cost control regulation of the hospital, and these medicines necessary for anti-DRTB treatment sometimes went short of supply in the hospital. Therefore, patients were often asked to buy medicines in pharmacies due to physicians’ fearing of violating the regulation, or the stock-out problem. Sometimes patients even had to use medical services from private providers, such as intravenous injection of drugs after being discharged from the designated hospital. Besides, DRTB patients often had co-morbidities, but the treatment costs for co-morbidities were not eligible for additional reimbursement or assistance. Additionally, expenditures that could be covered by medical assistance or supplemental reimbursement were often not directly deducted from the total cost, and patients needed to pay before they could get reimbursement. Some patients also admitted that they defaulted treatment due to financial difficulties.

**Table:** Box 5. Quotations on the financial burden of DRTB patients

*I was hospitalized in Ningxia Fourth hospital last year. I stayed in hospital for 90 days and had taken five times of fiberoptic bronchoscopy…I was enrolled in URBMI and paid over CNY 20,000 out-of-pocket. I am a farmer and I borrowed money to pay for the cost…(FGD, DRTB patient in Ningxia)* *Currently I do not need to pay out-of-pocket under the special policy for outpatient visit, but I was once hospitalized for liver damage, and I paid CNY 40–50,000 out-of-pocket for this hospitalization, and the actual reimbursement rate was only 60%…(FGD, DRTB patient in Zhejiang)* *I experienced shortage of medicines in hospitals, and I felt that the side-effects of domestic medicines were substantial and I bought medicines made in India…When I was hospitalized, some medicines were not covered by health insurance and they cost several thousands CNY…(FGD, DRTB patient in Zhejiang)* *I was hospitalized for 6 to 9 months in total, and I needed to pay over 100,000 CNY before I could get reimbursed… Sometimes there was a shortage of medicines in the hospital, I needed to go the pharmacies to buy medicines which were more expensive…The auxiliary medicines could were not covered by health insurance (FGD, DRTB patients in Jilin)* *I needed to go to private clinic for injection as the medicine was prescribed by the designated hospital, not the community health care centers, because the community health care centers would not help you inject the medicines not prescribed by them. I paid the private clinic 20 CNY each time for the injection…(FGD, DRTB patient in Jilin)* *I used up my money during hospitalization, and I did not take medicines after discharging from hospital…(FGD, DRTB patient in Ningxia)*	

*FGD* Focus-group discussion, *DRTB* Drug-resistant tuberculosis

## Discussions

Our results show that the quality of clinical services for screening, diagnosis, and treatment of DRTB patients significantly improved during the intervention period. Gains were especially large for JL where there was almost no standardized DRTB treatment and management prior to project implementation. However, the financial burden of DRTB patients was not remedied during the intervention period, likely due to the delays in implementing DRTB financing policies.

### Effective interventions and further challenges in improving the quality of DRTB clinical services

Our study findings align with existing research regarding the effectiveness of rapid DST technologies in improving DRTB case finding capacity. It is also notable that the supporting policies to guarantee the supply of reagents for assays and provide tests free-of-charge for patients have ensured the use of the new technologies in the screening for DRTB patients upon necessity. The shortened diagnosis timeline and strict referral policies also increased the treatment inclusion rate, as evidenced by other studies [5, 16, 31]. As for treatment inclusion and completion, the bi-directional referral policies and the supervision on the clinical practice of physicians could improve physicians’ awareness to enroll DRTB patients into standard treatment, and help standardize the clinical practice of health workers through regulation and mentoring. The centralized treatment for DRTB patients in prefecture-level designated hospitals may also reduce the likelihood of receiving sub-standard treatment in other institutions. Nevertheless, some studies in other low- and middle- income countries (LMICs) suggested that the decentralized care model performed better in DRTB treatment quality than centralized care [32–34], and the effects of bi-directional referral policies for DRTB clinical performance could hardly be examined separately in our research as the interventions were implemented as a package.

Despite of the improvements achieved, challenges remain in maintaining and further improving the quality of DRTB health services. Although the price for the GeneXpert test is still expensive and not covered by the health insurance, the government must ensure the sustainability of funds to maintain or further improve case detection capacity after project ends. The study on the use of GeneXpert in Ghana also mentioned about the concern of government budget to run GeneXpert [35]. For LMICs with high burden of TB, the high demand for testing reagents and the pooled purchase by the government could potentially lower the unit price and make it affordable for the government to cover the testing cost. As for treatment enrollment, quality and completion, 20–30% of patients still did not initiate standard treatment, and a considerable percentage of patients in ZJ in JL did not take culture tests at the end of 6 months’ treatment as required by the clinical guidelines. While the bi-directional referral policies and the regular supervision implemented during the project were both directed at regulating health providers’ behaviors, interventions aiming at encouraging DRTB patients to follow standard treatment were nearly nonexistent. Additionally, the contradictions between the clinical needs of DRTB treatment and the public health insurance regulations regarding the prescription of medicines should be resolved to ensure treatment quality. The shortage of second-line drug supply in less-developed regions also needs to be taken seriously and addressed through policy interventions.

### Challenges and potential policies to relieve the financial burden of DRTB patients

The universal occurrence of the CHE among the DRTB patients indicates that financial burden is still heavy under the current health insurance and medical assistance system in China. Failure to address this issue may become a bottleneck for further improving the quality of DRTB-related health services and treatment outcomes. As revealed in the qualitative study, the current benefit packages of public health insurance could hardly meet the clinical needs of DRTB patients due to the limited range of coverage regarding medicines and health services, and the actual reimbursement rate of treatment cost is very low. However, the fact that the new financing policies were not implemented on time indicates the difficulties in mobilizing financial resources to relieve the economic burden of patients, especially for the less-developed regions with relatively low government revenue and tight health insurance budgets. Moreover, the health insurance office is unwilling to increase the reimbursement rate for a specific disease for as they feel that it’s unfair for other patients.

In light of these challenges in pushing forward financing policies for DRTB patients, new strategies may be considered in line with the ongoing policy movement to end poverty in China. In recent years the Chinese government has focused on the national policy of targeted poverty-alleviation [36], and in our study the poorest 1/3 patients had much higher likelihood of incurring extremely high financial burden during treatment, which would in turn aggravate their poverty status. The specialized funding to support DRTB patients to complete treatment, especially for those poorest patients, could potentially be secured through the national targeted poverty alleviation program. Such a program should provide coverage for DRTB as well as comorbidities, which were extremely burdensome for DRTB patients. Additionally, as migrant patients may also have more difficulties accessing DRTB care and bear higher financial burden of treatment, though not explored in detail in this study but revealed in other studies [37–39], the assistance polices for DRTB policies may also cover migrant workers who are often excluded from social assistance policies without local registered residence (Hukou).

In addition to financing policies, cost containment is also crucial for reducing the financial burden and developing feasible financial support policies for DRTB patients. On the one hand, the unit-price of some tests and medicines are expensive, as evidenced in previous studies and our qualitative research [12]. The health insurance office may take the lead in the negotiation to lower the price for the necessary tests and medicines, as suggested before for the promotion of GeneXpert. On the other hand, the clinical practices need to be further standardized and avoid the providing unnecessary services due to profit-driven motivations, which has been widely discussed during the healthcare reform in China [40–42]. Our findings that patients in JL paid the highest OOP during the first 6 months and had the highest likelihood of incurring catastrophic expenditure could possibly be associated such motivations. Our qualitative research with directors and physicians from infectious disease hospitals and TB dispensaries in JL did reveal their concerns about the revenue from medical services which is related to their income. Physicians in the infectious department in general hospitals in other provinces also said that their department did not earn as much revenue as other departments, but there were some mechanisms to balance the income of physicians in the hospital. Nevertheless, the infectious disease hospitals or TB dispensaries in JL do not have such departments that usually generate higher income. In light of these findings, the motivations to earn income from medical services need to be altered for better control of DRTB patients’ financial burden. The case-based payment approach which sets a fixed payment amount for a single case based on a reasonable estimation of the appropriate treatment cost, could be a possible solution [43]. This is particularly important for governments in low- and middle-income countries with tight budgets to provide universal coverage for DRTB patients.

Although our findings provide useful policy implications, there are several limitations. First, the sample size for the examination of medical records examination is very small, and the representativeness of the sample could not be well-proved. Additionally, we only collected information on the treatment experiences and OOP for the first 6 months of treatment given feasibility constraints. Therefore, we did not examine medical expenditures for the whole treatment course. Recall bias was likely to happen as the many patients reported their experiences more than 1 year ago. Moreover, compared to the overall registered patients, the sample for patient survey included a lower percentage of migrant patients, and the financial burden may be under estimated for the overall patient group. Despite these limitations, we believe our findings on the quality of DRTB related clinical services are still sound, as results from medical records are similar to the those from registration data. The finding that heavy financial burden was still prevailing among DRTB patients were also unlikely to be influenced by the limitations with our rich evidence.

With regards to the challenges and potential strategies we identified in further improving the clinical quality and relieving the financial burden of DRTB patients, future projects may design interventions on key aspects such as promoting the optimal use of new DST technologies through sustainable funding, standardizing the clinical practices of physicians as well as developing impelling policies to provide financial protection and control treatment cost for DRTB patients, and evaluate the effectiveness of these interventions based on concrete evidence.

## Conclusions

The quality of DRTB related clinical services has significantly improved since the implementation of comprehensive interventions on the promotion of rapid DST technology and the regulation of clinical practices. Nevertheless, the high financial burden of DRTB patients has not been alleviated, primarily due to the delayed implementation of financing policies. Stronger political commitment and leadership are required to mobilize multiple financing resources to provide additional financial support to DRTB patients.

## Supplementary Information

**Additional file 1: Table S1.** Characteristics of DRTB patients registered in the TBIMS in 2015 and 2018. **Table S2.** Bacterially confirmed patients taking DST and molecular DST in 2015 and 2018. **Table S3.** The number and percentage of DRTB patients diagnosed, included in treatment and under treatment at the end of 6 months’ treatment. **Table S4.** DRTB patients under treatment and taking culture test at the end of 6 months’ treatment. **Table S5.** Results of onsite examination of medical records. **Table S6.** Characteristics of DRTB patients who reported and who did not report OOP.

## Data Availability

The datasets generated and analyzed during the current study are not publicly available due to the regulations of China CDC. Readers of the article need to discuss with China CDC and obtain their permission before the release of the dataset.

## Abbreviations

DRTB: Drug-resistant tuberculosis
WHO: World Health Organization
RRTB: Rifampicin-resistant tuberculosis
MDRTB: Multi-drug resistant tuberculosis
UEBMI: Urban Employee Basic Medical Insurance
URBMI: Urban Resident Basic Medical Insurance
NCMS: New Cooperative Medical Schemes
DST: Drug susceptibility test
OOP: Out-of-pocket
NHC: National Health Commission
ZJ: Zhejiang Province
JL: Jilin Province
NX: Ningxia Hui Autonomous Region
TBIMS: Tuberculosis Information Management System
FGD: Focus-group discussion
CDC: Center for Disease Control and Prevention
LIMCs: Low- and middle-income countries
NCD: Non-communicable disease
