# Supplementary material for: Impacts of a comprehensive tuberculosis control model on the quality of clinical services and the financial burden of treatment for patients with drug-resistant tuberculosis in China: a mixed-methods evaluation
Source: Infect Dis Poverty. 2021 Apr 21;10:54. doi: 10.1186/s40249-021-00832-5 (PMC8059277; doi:10.1186/s40249-021-00832-5)
Supplement: Supplementary file 1 — Additional file 1: Table S1. Characteristics of DRTB patients registered in the TBIMS in 2015 and 2018. Table S2. Bacterially confirmed patients taking DST and molecular DST in 2015 and 2018. Table S3. The number and percentage of DRTB patients diagnosed, included in treatment and under treatment at the end of 6 months’ treatment. Table S4. DRTB patients under treatment and taking culture test at the end of 6 months’ treatment. Table S5. Results of onsite examination of medical records. Table S6. Characteristics of DRTB patients who reported and who did not report OOP. [file 40249_2021_832_MOESM1_ESM.docx]

**Supplemental Materials**

Table 1: Characteristics of DRTB patients registered in the TBIMS in 2015 and 2018

|  | TBIMS 2015 | | TBIMS 2018 | |
| --- | --- | --- | --- | --- |
|  | N | % | N | % |
| Total | 766 |  | 1765 |  |
| Province |  |  |  |  |
| ZJ | 552 | 72.1 | 661 | 37.5 |
| JL | 141 | 18.4 | 1015 | 57.5 |
| NX | 73 | 9.5 | 89 | 5.0 |
| Gender |  |  |  |  |
| Female | 208 | 27.2 | 481 | 27.3 |
| Male | 558 | 72.8 | 1284 | 72.7 |
| Age |  |  |  |  |
| <30 | 162 | 21.1 | 315 | 17.8 |
| 30-59 | 417 | 54.4 | 949 | 53.8 |
| >59 | 187 | 24.4 | 501 | 28.4 |
| Ethnicity |  |  |  |  |
| Han | 701 | 91.5 | 1684 | 95.4 |
| Other | 65 | 8.5 | 81 | 4.6 |
| Marriage |  |  |  |  |
| Married |  |  |  |  |
| Current place of residence |  |  |  |  |
| in the county of registration | 384 | 50.1 | 1193 | 67.6 |
| in the prefecture of registration,  but not the county | 159 | 20.8 | 273 | 15.5 |
| in the province of registration,  but not the prefecture | 60 | 7.8 | 103 | 5.8 |
| outside the province of registration | 163 | 21.3 | 196 | 11.1 |

Table 2: Bacterially confirmed patients taking DST and molecular DST in 2015 and 2018

| Province | 2015 | | | | | 2018 | | | | | P1 | P 2 |
| --- | --- | --- | --- | --- | --- | --- | --- | --- | --- | --- | --- | --- |
|  | No. of bacterially confirmed TB  patients | taking DST | | taking molecular DST among tested | | No. of bacterially confirmed TB  patients | taking DST | | taking molecular DST among tested | |  |  |
|  |  | N | % | N | % |  | N | % | N | % |  |  |
| ZJ | 11016 | 6323 | 57.4 | 1005 | 15.9 | 14402 | 13486 | 93.6 | 10759 | 79.8 | <0.001 | <0.001 |
| JL | 4468 | 560 | 12.5 | 258 | 46.1 | 6409 | 5542 | 86.5 | 4368 | 78.8 | <0.001 | <0.001 |
| NX | 880 | 261 | 29.7 | 38 | 14.6 | 1260 | 1152 | 91.4 | 1117 | 97.0 | <0.001 | <0.001 |

(P1 is for the chi-square test of the percentage of patients taking DST in 2015 and 2018, P2 is for the chi-square test of the percentage of patients taking molecular DST among those tested in 2015 and 2018)

Table 3: The number and percentage of DRTB patients diagnosed, included in treatment and under treatment at the end of 6 months’ treatment

| Province | 2015 | | | | | 2018 | | | | | P1 | P2 |
| --- | --- | --- | --- | --- | --- | --- | --- | --- | --- | --- | --- | --- |
|  | No. of diagnosed patients | included in treatment | | under treatment at the end of 6-months' treatment | | No. of diagnosed patients | included in treatment | | under treatment at the end of 6-months' treatment | |  |  |
|  |  | N | % | N | % |  | N | % | N | % |  |  |
| ZJ | 552 | 258 | 46.7 | 234 | 90.7 | 661 | 494 | 74.7 | 475 | 96.2 | <0.001 | 0.002 |
| JL | 141 | 38 | 27.0 | 21 | 55.3 | 1015 | 747 | 73.6 | 687 | 92.0 | <0.001 | <0.001 |
| NX | 73 | 39 | 53.4 | 33 | 84.6 | 89 | 73 | 82.0 | 55 | 75.3 | <0.001 | 0.255 |

(P1 is for the chi-square test of the percentage of diagnosed DRTB patients included in treatment in 2015 and 2018, P2 is for the chi-square test of the percentage of patients under treatment at the end of 6-months' treatment among those treated in 2015 and 2018)

Table 4: DRTB patients under treatment and taking a culture test at the end of 6 months’ treatment

| Province | 2015 | | | | | 2018 | | | | | P1 | P2 |
| --- | --- | --- | --- | --- | --- | --- | --- | --- | --- | --- | --- | --- |
|  | No. under  treatment at the end of 6-months' treatment | taking culture test at the end of 6-months' treatment | | Negative cases | | No. under  treatment at the end of 6-months' treatment | taking culture test at the end of 6-months' treatment | | Negative cases | |  |  |
|  |  | N | % | N | % |  | N | % | N | % |  |  |
| ZJ | 234 | 174 | 74.4 | 162 | 93.1 | 475 | 357 | 75.2 | 344 | 96.4 | 0.818 | 0.096 |
| JL | 21 | 2 | 9.5 | 0 | 0.0 | 687 | 280 | 40.8 | 202 | 72.1 | 0.004 | 0.024 |
| NX | 33 | 17 | 51.5 | 13 | 76.5 | 55 | 50 | 90.9 | 45 | 90.0 | <0.001 | 0.158 |

(P1 is for the chi-square test of the percentage of DRTB patients under treatment at the end of 6-months' treatment who took culture test in 2015 and 2018, P2 is for the chi-square test of the percentage of negative cases among those treated in 2015 and 2018)

Table 5: Results of onsite examination of medical records

| Province | Total cases | under treatment at the end of the 6th month | | No. of cases under treatment  in local hospital | Culture test at the end of 6th month | | No. of  cases with results | Negative cases | |
| --- | --- | --- | --- | --- | --- | --- | --- | --- | --- |
|  |  | N. | % |  | N. | % |  | N | % |
| ZJ | 24 | 21 | 87.5 | 18 | 16 | 88.9 | 12 | 12 | 100.0 |
| JL | 58 | 52 | 89.7 | 52 | 22 | 42.3 | 18 | 15 | 83.3 |
| NX | 22 | 19 | 86.4 | 19 | 12 | 63.2 | 12 | 12 | 100.0 |
| Total | 104 | 92 | 88.5 | 89 | 50 | 56.2 | 42 | 39 | 92.9 |

(Note: the culture test usually takes 1-2 months, and the results of some patients had not yet come out at the time of final evaluation)

Table 6: Characteristics of DRTB patients who reported and who did not report OOP

|  | OOP reported | | OOP missing | | P of Chi-square test |
| --- | --- | --- | --- | --- | --- |
|  | N | % | N | % |  |
| Total | 147 |  | 57 |  |  |
| Province |  |  |  |  |  |
| ZJ | 32 | 21.8 | 7 | 12.3 | 0.111 |
| JL | 80 | 54.4 | 40 | 70.2 |  |
| NX | 35 | 23.8 | 10 | 17.5 |  |
| Gender |  |  |  |  |  |
| Female | 34 | 23.1 | 21 | 36.8 | 0.048 |
| Male | 113 | 76.9 | 36 | 63.2 |  |
| Age |  |  |  |  |  |
| <30 | 24 | 17.3 | 5 | 10.0 | 0.361 |
| 30-59 | 83 | 59.7 | 35 | 70.0 |  |
| >59 | 32 | 23.0 | 10 | 20.0 |  |
| Ethnicity |  |  |  |  |  |
| Han | 19 | 13.7 | 8 | 16.0 | 0.686 |
| Marriage |  |  |  |  |  |
| Married | 120 | 86.3 | 42 | 84.0 | 0.479 |
| Current place of residence |  |  |  |  |  |
| in the county of registration | 129 | 87.8 | 50 | 87.7 | 0.479 |
| in the prefecture of registration, but not the county | 10 | 6.8 | 2 | 3.5 |  |
| outside the province of registration | 8 | 5.4 | 5 | 8.8 |  |
| Type of registered residence |  |  |  |  |  |
| rural | 87 | 59.2 | 34 | 59.7 | 0.952 |
| Education level |  |  |  |  |  |
| Primary school and below | 44 | 29.9 | 22 | 38.6 | 0.036 |
| Junior high school | 46 | 31.3 | 20 | 35.1 |  |
| Senior high school and the equivalent | 28 | 19.1 | 13 | 22.8 |  |
| College and above | 29 | 19.7 | 2 | 3.5 |  |
| Insurance coverage |  |  |  |  |  |
| UEBMI | 28 | 19.1 | 10 | 17.5 | 0.851 |
| NCMS | 65 | 44.2 | 28 | 49.1 |  |
| RBMI | 45 | 30.6 | 17 | 29.8 |  |
| Other | 9 | 6.1 | 2 | 3.5 |  |
| Average household income |  |  |  |  |  |
| lowest 1/3 | 42 | 29.0 | 25 | 45.5 | 0.080 |
| middle 1/3 | 50 | 34.5 | 16 | 29.1 |  |
| highest 1/3 | 53 | 36.6 | 14 | 25.5 |  |
| NCD status |  |  |  |  |  |
| With other NCD | 54 | 36.7 | 22 | 38.6 | 0.805 |
| Inpatient service use |  |  |  |  |  |
| Yes | 140 | 95.2 | 52 | 91.2 | 0.275 |
